# Parent smoker role conflict and planning to quit smoking: a cross-sectional study

**DOI:** 10.1186/1471-2458-13-164

**Published:** 2013-02-22

**Authors:** Joan Friebely, Nancy A Rigotti, Yuchiao Chang, Nicole Hall, Victoria Weiley, Janelle Dempsey, Bethany Hipple, Emara Nabi-Burza, Sybil Murphy, Heide Woo, Jonathan P Winickoff

**Affiliations:** 1 Center for Child and Adolescent Health Research and Policy, Massachusetts General Hospital, Boston, MA, USA; 2 Pediatric Research in Office Settings, American Academy of Pediatrics, Elk Grove Village, IL, USA; 3 General Medicine Division, Massachusetts General Hospital, Boston, MA, USA; 4 AAP Richmond Center of Excellence, American Academy of Pediatrics, Elk Grove Village, IL, USA; 5 Tobacco Research and Treatment Center, Massachusetts General Hospital, Boston, MA, USA

**Keywords:** Parent smoker identity, Parent smoker role conflict, Tobacco smoke exposure, Readiness to quit, Stages of change, Tobacco control, Pediatrics, Smoking cessation, Parent child dyad

## Abstract

**Background:**

Role conflict can motivate behavior change. No prior studies have explored the association between parent/smoker role conflict and readiness to quit. The objective of the study is to assess the association of a measure of parent/smoker role conflict with other parent and child characteristics and to test the hypothesis that parent/smoker role conflict is associated with a parent’s intention to quit smoking in the next 30 days. As part of a cluster randomized controlled trial to address parental smoking (Clinical Effort Against Secondhand Smoke Exposure—CEASE), research assistants completed exit interviews with 1980 parents whose children had been seen in 20 Pediatric Research in Office Settings (PROS) practices and asked a novel identity-conflict question about “how strongly you agree or disagree” with the statement, “My being a smoker gets in the way of my being a parent.” Response choices were dichotomized as “Strongly Agree” or “Agree” versus “Disagree” or “Strongly Disagree” for the analysis. Parents were also asked whether they were “seriously planning to quit smoking in 30 days.” Chi-square and logistic regression were performed to assess the association between role conflict and other parent/children characteristics. A similar strategy was used to determine whether role conflict was independently associated with intention to quit in the next 30 days.

**Methods:**

As part of a RTC in 20 pediatric practices, exit interviews were held with smoking parents after their child’s exam. Parents who smoked were asked questions about smoking behavior, smoke-free home and car rules, and role conflict. Role conflict was assessed with the question, “Please tell me how strongly you agree or disagree with the statement: ‘My being a smoker gets in the way of my being a parent.’ (Answer choices were: “Strongly agree, Agree, Disagree, Strongly Disagree.”)

**Results:**

Of 1980 eligible smokers identified, 1935 (97%) responded to the role-conflict question, and of those, 563 (29%) reported experiencing conflict. Factors that were significantly associated with parent/smoker role conflict in the multivariable model included: being non-Hispanic white, allowing home smoking, the child being seen that day for a sick visit, parents receiving any assistance for their smoking, and planning to quit in the next 30 days. In a separate multivariable logistic regression model, parent/smoker role conflict was independently associated with intention to quit in the next 30 days [AOR 2.25 (95% CI 1.80-2.18)].

**Conclusion:**

This study demonstrated an association between parent/smoker role conflict and readiness to quit. Interventions that increase parent/smoker role conflict might act to increase readiness to quit among parents who smoke.

**Trial registration:**

Clinical trial registration number: NCT00664261.

## Background

Tobacco smoke exposure (TSE) endangers the health of infants and children and leads to increased risk of asthma, other respiratory diseases, otitis media, cognitive dysfunction, neurobehavioral disorders, and sudden infant death syndrome [1-3]. Tobacco smoke has “sticky” properties and very small particulate matter that prevent smokers from completely protecting others in close proximity from its harms [4]. Parental smoking is associated with parental morbidity and mortality, increased risk of house fires, diversion of income, and greater likelihood of children’s tobacco addiction [5]. In these ways, smoking cigarettes attenuates parents’ performance of the protective function that is a defining characteristic of the parental role [6].

The child health care setting provides unique teachable moments to motivate parents to quit smoking as a result of the number of contacts a parent has with his or her child’s healthcare provider and the strong link between TSE and chronic and acute childhood illness [5,7-9]. The child’s wellness visit may be the only opportunity for many parents to access physician assisted smoking cessation resources due to the steadily increasing rate of uninsured adults [7]. Child healthcare clinicians, through the schedule of primary care visits, are often in a position to intervene with parental smokers in a repeated and consistent manner over the course of many years [5]. For parents who lack a primary care provider, their child’s doctor may be a key access point for pharmacological advice and treatment for tobacco addiction [10,11].

Having parents’ smoking addressed in child healthcare visits by their children’s clinicians may prompt parents to experience conflict between their parent and smoker identities. Identity, especially identity in relation to others with in a social group, is developed through processes of reflection and categorization; identity may contain several different roles, including the role of parent and the role of smoker [12]. The role of ‘parent’ evokes the parents’ own expectations of their actions and behavior as well as the expectations of others within society as a whole. Role conflict is particularly apparent in smoking parents because the roles of parent and smoker can have incompatible behavioral standards and social expectations. Role conflict, as defined by tension among identities, has been identified as a major force in the motivational process [13,14]. The discomfort associated with role conflict might enhance motivation to quit smoking. To our knowledge, this idea has not been examined previously.

To explore the extent and nature of conflicts that may occur between the parental role and smoking, we measured the role conflict experienced by parents who smoked at baseline from data collected in a randomized controlled trial of tobacco cessation service delivery at child healthcare visits. We assessed the parent and child characteristics associated with the parent/smoker role conflict variable. We were particularly interested in testing the hypothesis that parent/smoker role conflict was associated with a parent’s intention to quit smoking in the next month. We chose this variable because planning to quit smoking within the next 30 days is the most advanced “stage of change” prior to action in the transtheoretical model of change [15]and has been an independent predictor of smoking cessation in longitudinal studies [16].

## Methods

Twenty practices were recruited from Pediatric Research in Office Settings (PROS), the practice-based research network of the American Academy of Pediatrics. Practices were randomized with 10 to the intervention arm and 10 to the control arm. The study participants were smoking parents who accompanied a child to an office visit. Eligibility criteria specified that participants be a parent or legal guardian of the child, over age 18, and English speaking, as well as having reported: “smoked a cigarette, even a puff, in the past 7 days.” The data for this analysis was collected as part of a larger study testing a pediatric-office based tobacco control program for families called CEASE (Clinical Effort Against Secondhand Smoke Exposure). The program includes training and materials to support the establishment of smoke-free home and car rules and smoking cessation, including connecting smoking parents to free cessation services and pre-printed nicotine replacement therapy prescription pads. The study protocol was approved by the Institutional Review Boards of the American Academy of Pediatrics and Massachusetts General Hospital.

### Participant enrollment

At each control and intervention practice, one or more research assistants were stationed at the exit and administered a screening questionnaire to all adults after their child’s visit. If the parent was eligible, the research assistant obtained informed consent. Parents were offered enrollment into the study after the completion of a baseline screening questionnaire. Parents who enrolled received 5 US dollars for completing the survey. Screening continued until 100 parents had been enrolled at each practice. Data collection was conducted after the introduction of the intervention; parents seen in invention practices may have been exposed to various aspects of the intervention, including educational materials such as posters and handouts, practice materials asking about smoking, and/or assistance in cessation from clinicians.

### Measures

Screening questions assessed parental smoking status (“Have you smoked a cigarette, even a puff, within the past 7 days?”), readiness to quit smoking (“Are you seriously planning to quit smoking within the next 30 days?”), demographic factors (parent’s age, gender, race and ethnicity, and level of education), age of the youngest child at the visit, the reason for the visit (routine, sick, follow-up, or other), and child’s insurance status (private insurance, Medicaid, self-pay, or other).

Parents who enrolled in the study also completed an enrollment survey that asked questions about recent quit attempts (“During the past 3 months, have you stopped smoking for more than one day because you were trying to quit smoking?”) and frequency of smoking (“Do you now smoke cigarettes every day or some days?”). Parents who smoked were categorized as daily or nondaily smokers and an average number of cigarettes smoked daily per 30 days was computed. The enrollment survey also asked whether parents received assistance for cessation (“During your visit today, did a doctor, nurse, or other health care provider discuss medicine to help you quit smoking, for example, nicotine replacement gum, patch or lozenge, or other medicine”) or (“discuss methods and strategies, other than medication, to help you quit smoking?”), and (“During your visit today, did anyone suggest you use a telephone ‘Quitline’ or other program to help you quit smoking?”). Enrolled parents were read statements of rules about smoking in the home. If the response choice was “No one is allowed to smoke anywhere,” the parent was categorized as having a strict rule against smoking in the home.

Role conflict was assessed with the question, “Please tell me how strongly you agree or disagree with the statement: ‘My being a smoker gets in the way of my being a parent.’ (Answer choices were: “Strongly agree, Agree, Disagree, Strongly disagree.”) The concept was adapted from a study [17] that used five questions to separately assess parent identity and smoker identity. For this study, it was not possible to use all of the questions from the previous study due to the length of the survey.

Anecdotal evidence from survey respondents revealed that parents had different interpretations of the question. Some interpreted it as referring to conflict between smoking and parental duties (also known as roles) while others interpreted it as referring to conflict in one’s perception or self-definition (identity) as a parent. For the purpose of this paper, the term ‘role’ will be used. Qualitative work would have to be undertaken to clarify how respondents understood the question as it relates to their role or their identity.

### Statistical analysis

The primary variable of interest was parent/smoker role conflict. For analysis, responses were dichotomized as “Strongly Agree” or “Agree” versus “Disagree” or “Strongly Disagree”. Using bivariate analysis, we examined the association between role conflict and other parent/children characteristics assessed in the baseline survey. These included the child’s age, insurance, and reason for the visit, and the parent’s age, sex, race, education, smoking behavior (daily vs. nondaily, cigarettes smoked per month, quit attempt within the past 3 months, and intention to quit in the next 30 days), home smoking policy, and report of whether they had received smoking cessation assistance from the child healthcare provider at the visit. To better describe the role conflict variable, variables that were significantly associated with role conflict at the p ≤ 0.10 level were entered into a multivariable logistic regression model to determine what factors were associated with conflict. An analogous strategy was used to determine whether role conflict was independently associated with intention to quit in the next 30 days. Both bivariate and multivariable analyses were conducted using logistic regression models with Generalized Estimating Equations (GEE) techniques to account for physician clustering. Adjusted Odds Ratio (aOR) with 95% Confidence Interval (CI) and p values are reported for all multivariable analyses. A two-tailed p < .05 was considered statistically significant. All analyses were conducted using SAS version 9.3 (The SAS Institute, Inc, Cary NC).

## Results

Out of the 1980 parents who were enrolled in the 10 control and 10 intervention practices, 1935 had complete data on parent/smoker role conflict and intention to quit in the next 30 days. Table 1 displays the characteristics of the sample. Most (73%) of the parents were 25 years or older, female (78%), non-Hispanic white (67%), had no more than a high school education (61%), smoked cigarettes daily (85%), and smoked 10 or more cigarettes per day (56%). Nearly half (46%) of parents reported that they had tried to quit smoking within the previous 3 months and 43% were seriously planning to quit smoking within the next 30 days. Almost one-quarter (23%) of parents reported having received assistance for tobacco cessation during the visit. Most of the children were 10 years old or less (86%) and insured by Medicaid (67%). Similar numbers of children were being seen for well visits (43%) and sick visits (41%); 17% of the visits were follow up visits.

**Table 1:** **Parent**/**smoker identity conflict by baseline characteristics of parents**, **children and childcare visits**

***Characteristic***	*“My being a smoker gets in the way of my being a parent.” *N (%*)			***Chi square***
	**Agree**	**Disagree**	**All**	***P Value***
	**(N = 563)**	**(N = 1372)**	**(N = 1935)**	
**Age**				0.006
<25	125 (22)	389 (28)	514 (27)	
> = 25	438 (78)	982 (72)	1420 (73)	
**Sex**				0.36
Male	115 (20)	300 (22)	415 (21)	
Female	447 (79)	1071 (78)	1518 (78)	
**Racial category**				0.003
Hispanic (any race)	65 (12)	149 (11)	214 (11)	
Mixed race	8 (1)	45 (3)	53 (3)	
Non-Hispanic Black	64 (11)	233 (17)	297 (15)	
Non-Hispanic Asian	5 (1)	4 (0)	9 (0)	
Native American, Pacific Islander	17 (3)	27 (2)	44 (2)	
Non-Hispanic White	399 (71)	904 (66)	1303 (67)	
Unknown	5 (1)	10 (1)	15 (1)	
**Education**				0.39
<High school	73 (13)	222 (16)	295 (15)	
High school graduate/GED	266 (47)	626 (46)	892 (46)	
Some college/trade school	167 (30)	377 (27)	544 (28)	
College graduate	55 (10)	142 (10)	197 (10)	
**Daily smoker**				0.043
Yes	492 (87)	1150 (84)	1642 (85)	
No	71 (13)	221 (16)	292 (15)	
# **Cigarettes**/**Day**				0.015
<10 cigarettes/day	214 (38)	613 (45)	827 (43)	
> = 10 cigarettes/day	345 (61)	748 (55)	1093 (56)	
**Recent 24**-**hour quit**				0.006
Yes	287 (51)	608 (44)	895 (46)	
No	273 (48)	756 (55)	1029 (53)	
**Home smoking rules**				0.058
No smoking anywhere	309 (55)	824 (60)	1133 (59)	
Smoking allowed some places/times	198 (35)	390 (28)	588 (30)	
Smoking allowed anywhere	50 (9)	137 (10)	187 (10)	
**Stage of change for quitting**				<0.0001
Seriously planning to quit in 30 days	326 (58)	508 (37)	834 (43)	
Considering quitting in 6 months	148 (26)	382 (28)	530 (27)	
Not considering quitting in 6 months	74 (13)	433 (32)	507 (26)	
**Child**’**s age**				0.033
<1 year	133 (24)	380 (28)	513 (27)	
1-5 years	226 (40)	575 (42)	801 (41)	
6-10 years	126 (22)	216 (16)	342 (18)	
>10 years	76 (13)	184 (13)	260 (13)	
**Child**’**s insurance coverage**				0.63
Medicaid	368 (65)	925 (67)	1293 (67)	
Private insurance/HMO	131 (23)	332 (24)	463 (24)	
Self-pay	17 (3)	33 (2)	50 (3)	
Other	42 (7)	73 (5)	115 (6)	
**Visit type**				0.0006
Routine visit	200 (36)	628 (46)	828 (43)	
Sick visit	253 (45)	532 (39)	785 (41)	
Follow-up	109 (19)	211 (15)	320 (17)	
**Received assistance for quitting at the visit**				
Yes	170 (30)	282 (21)	452 (23)	
No	393 (70)	1090 (79)	1483 (77)	0.001

* Not all categories add up to 100% due to missing responses.

Twenty-nine percent of parents (n = 563) said that they experienced parent/smoker conflict. In bivariate analyses (Table 1), parents who experienced parent/smoker role conflict were more likely to be older, non-Hispanic white, smoke more heavily, made a recent quit attempt, planned to quit in the next month, accompanied a child over 5 years old, visited the pediatrician because their child was sick, and received assistance for smoking cessation during that visit (p < 0.05). Parental gender, education, and child’s health insurance were not associated with parent/smoker role conflict.

In the multivariable logistic model, factors that were significantly associated with parent/smoker role conflict included: parent age 25 or older, having made a recent quit attempt, allowing smoking somewhere inside the home or at some times, having a sick child or other type of visits as opposed to a routine visit, and receiving any assistance for tobacco dependence treatment. Parents who reported being non-Hispanic black or mixed race were less likely to report the parent/smoker identity conflict compared to non-Hispanic white (see Table 2).

**Table 2:** **Factors associated with parent**/**smoker identity conflict**: **multivariable logistic regression analysis**

	***Adjusted odds ratio*** (***95% CI***)	***P value***
Parent Age ≥ 25	1.32 (1.01, 1.72)	<0.0001
Race (ref: non-Hispanic White)		
Non-Hispanic Black	0.61 (0.43, 0.87)	0.006
Mixed race	0.37 (0.18, 0.77)	0.007
Hispanic	1.10 (0.76, 1.60)	0.62
Asian	3.50 (0.68, 18.02)	0.13
Recent 24-hr quit attempt	1.64 (1.31, 2.06)	<0.0001
Home smoking rules (ref: no one allowed anywhere)		
Permitted some places or at some times	1.41 (1.08, 1.86)	0.013
Permitted anywhere	0.95 (0.62, 1.45)	0.80
Visit Type (Ref: routine visit)		
Sick Child	1.49 (1.20, 1.86)	<0.001
Other visit type	1.48 (1.06, 2.06)	0.020
Received assistance for quitting at the visit	1.74 (1.33, 2.28)	<0.0001

*Daily smoking status, # cigarettes/day, and age of the child were entered in the model but all were non-significant.

In a separate multivariate logistic model, parent/smoker role conflict was independently associated with intention to quit in the next 30 days [aOR 2.25 (95% CI 1.80-2.18)] (Table 3). Other factors that were associated with planning to quit smoking in that model included having made a quit attempt within the previous 3 months, receiving cessation assistance at the office visit, being age 25 years or older, and being a nondaily smoker.

**Table 3:** **Factors associated with planning to quit in the next 30 days**: **multivariable logistic regression analysis**

	***Adjusted odds ratio*** (***95% CI***)	***P value***
Identity conflict agree vs. disagree	2.25 (1.80-2.81)	<0.0001
Recent 24-hr quit	2.63 (2.14-3.23)	<0.0001
Nondaily smoking vs. daily smoking	1.68 (1.20-2.36)	0.003
Parent age >24 years vs. ≤ 24 years	1.42 (1.15-1.75)	0.001
Assistance	1.93 (1.45-2.57)	<0.0001

*# Cigarettes/Day was entered in the model but was not significant.

## Discussion

In this study, 29% of parents who smoked and were visiting a pediatrician with their child reported experiencing role conflict, as measured by the statement, “Being a smoker gets in the way of being a parent.” Parents who reported this conflict were more likely to be planning to quit smoking in the near future, and the association persisted even after an analysis adjusting for other factors associated with planning to quit.

Overall, we found that 43% of parents reported that they seriously planned to quit smoking within the next 30 days, and 46% had attempted to quit smoking within the previous 3 months. Compared with studies of stages of change in other populations, this percentage is high: about 20% of smokers typically qualify for the preparation level of readiness to quit [18]. Enhanced readiness to quit suggests an even greater opportunity for pediatric healthcare providers to influence smokers compared to adult healthcare providers or smoking cessation programs for the general population. Having a recent quit attempt had the strongest association with planning to quit smoking within the next 30 days [aOR 2.63(95% CI 2.14-3.23)]. Being a nondaily smoker was also associated with readiness to quit. This finding is consistent with a study of light smokers in which nondaily smokers differed from daily smokers in having more recent and planned quit attempts [19].

In addition, receipt of cessation assistance for tobacco dependence from the child healthcare provider was associated with planning to quit smoking in the next 30 days. However, because all data for the current study were gathered after the child healthcare visit, it is impossible to say whether assistance may have increased the likelihood of being ready to quit or whether being ready to quit may have elicited assistance from clinicians able to provide it.

Few studies about the distribution of smokers by stage of change find that age is associated with readiness to quit [20]. This study’s concentration of parents may skew the impact of age on our findings and account for the association with older parents. Jarvis [21] reported that among women, there was a linear increase in the odds of cessation with each additional child, and this effect was marginally present in men, as well. It is likely age 25 is associated with a higher number of children in our study population. We could not examine the interaction of number of children and intention to quit because we did not collect data on the number of children per family unit. Cessation may have been preceded by intention to quit within the next 30 days, as reflected in our data.

We explored the question of parent/smoker role conflict out of our ultimate interest in tailoring interventions for parents accompanying children to child healthcare visits. Women and men often designate “parent” as their most important identity or role [22].We speculated that the hierarchical position of the parental versus smoker role could invoke protective instincts to increase readiness to quit smoking. Theories of motivational intervention support the concept that when adults are confronted with a discrepancy between their perceived versus desired roles (smoking parent versus non-smoking parent), their motivation can be increased through a brief motivational moment. Physicians can emphasize the behaviors that lead to such a discrepancy and the potential solutions to resolve it. Increased motivation prompted by role conflict is consistent with predictions of self-discrepancy theory, which states that people compare themselves to internalized standards and are motivated to reduce the gap in order to remove discrepancy in self-guides. Sick visit type, planning to quit in the next 30 days, allowing smoking within the home, and receiving assistance for tobacco dependence were all associated with experiencing conflict. These findings are associative but might suggest possible parent/smoker role mechanisms that could be utilized in future studies to enhance motivation to quit.

### Limitations of the study

A limitation of the study is that parent/smoker role conflict was measured with a single question that was derived from a previously published scale [17]. A second limitation is that analyses were cross-sectional; causal inferences cannot be made about the association of the conflict variable with readiness to change. A further limitation is that the majority of the parents surveyed were white, non-Hispanic. These limitations will be addressed in future work.

## Conclusions

Interventions that increase parent/smoker role conflict might increase motivation to quit among parents who smoke. Physicians can utilize the child’s visit to initiate brief motivational moments with parents to both emphasize these conflicts and also provide an action-oriented approach to resolve them. However, not all types of motivation to quit are equally associated with successful cessation. A recent report in which motivation to quit was deconstructed into desire, duty and intention found that duty was negatively associated with success, unless it resulted in the smoker desiring to quit [23]. Duty was measured by response to the statement, “I ought to stop smoking,” as opposed to “I want to stop smoking” and “I intend to quit smoking.” Future qualitative work is needed to understand what the parent/smoker role question means to parents and how it might influence their tobacco use.

## Abbreviations

TSE: Tobacco smoke exposure; AOR: Adjusted odds ratio; CI: Confidence interval; PROS: Pediatric Research in Office Settings

## Competing interests

None of the authors have any financial or non-financial competing interests to disclose.

## Authors’ contributions

Dr. JF conceived of and designed this paper, drafted the manuscript and revised it, and takes full responsibility for the final submission; Dr. YC advised on and conducted data analyses and participated in the interpretation of results; Dr. NR made substantial contributions to the conception and design, and revised it critically for important intellectual content; Dr. JPW conceived of and conducted the larger trial as the Principal Investigator, made substantial contributions to the conception and design, and revised it critically for important intellectual content; all other co-authors (NH, VW, JD, BH, EN, SM, and HW) had full access to all of the data in the study and made substantial intellectual contributions to the conception and design, analysis and interpretation of data, editing the manuscript, and approving the final version for publication. All authors read and approved the final manuscript.

## Pre-publication history

The pre-publication history for this paper can be accessed here:

http://www.biomedcentral.com/1471-2458/13/164/prepub
